# Identification of two distinct hereditary hemorrhagic telangiectasia patient subsets with different hepatic perfusion properties by combination of contrast-enhanced ultrasound (CEUS) with perfusion imaging quantification

**DOI:** 10.1371/journal.pone.0215178

**Published:** 2019-04-11

**Authors:** Roland C. Schelker, Kornelia Andorfer, Franz Putz, Wolfgang Herr, Ernst-Michael Jung

**Affiliations:** 1 Department of Internal Medicine III, Hematology & Oncology, University Hospital of Regensburg, Regensburg, Germany; 2 Department of Ear-Nose-Throat, University Hospital of Regensburg, Regensburg, Germany; 3 Department of Nephrology, University Hospital of Regensburg, Regensburg, Germany; 4 Department of Radiology, University Hospital of Regensburg, Regensburg, Germany; Texas A&M University, UNITED STATES

## Abstract

**Background:**

Hereditary hemorrhagic telangiectasia (HHT) is marked by arteriovenous fusion comprising hepatic vascular malformations (HVaMs) with the chance of bleeding.

**Aims:**

We investigated HVaMs in HHT patients by combination of contrast-enhanced ultrasound (CEUS) with perfusion imaging quantification to be able to sub-classify a high risk cohort of asymptomatic HHT patients.

**Methods:**

The imaging characteristics on CEUS in 34 patients (aged 21–84 years; mean 58.9) with HHT were retrospectively evaluated. Real-time contrast harmonic imaging, sulfur hexafluoride-filled microbubbles and motion adjustment were utilized. Cine loops of the liver were digital stored, perfusion was quantified using a software reading DICOM data`s.

**Results:**

HVaMs were diagnosed in 31 out of 34 patients. Significant uppermost peak enhancement (PE), wash-in area under the curve (WiAUC) and wash-in perfusion index (WiPI) were identified in the shunt region (100%), next in the hilar region (PE 32.6%; WiAUC 33.9%; WiPI 34.1%), and the lowest in the hepatic parenchyma (PE 10.2%; WiAUC 12.0%; WiPI 9.5%). The perfusion parameters in the shunt region compared to the other regions were significantly increased in one subgroup of patients. Consistent with this, the intrahepatic portal vein diameter and Buscarini grading was significantly higher, while portal vein peak velocity was significantly lower in this patient subset. By statistical analysis, we could correlate PE and WiPI to these clinical parameters, while WiAUC showed no clinical association.

**Conclusions:**

For the first time we combined CEUS findings with motion adjustment software to quantitative determine perfusion parameters of a cohort of HHT patients. Hereby, we could identify a subset of HHT patients with two markedly increased parameter values in the shunt region compared to the hilus/hepatic parenchyma. This could contribute to sub-classify a high-risk group of HHT patients with therapeutic indication.

## Introduction

Hereditary hemorrhagic telangiectasia (HHT) is a rare autosomal dominantly transmitted disorder affecting small mucocutaneous blood vessels and/or the vasculature of various viscera [1, 2].

Hepatic vascular malformations (HVaMs) represent the most frequent type of visceral involvement, demonstrated in approximately 78% of HHT patients, however only 8% being symptomatic [3, 4]. The clinical manifestation is most commonly represented by high-output heart failure and/or portal hypertension [5]. HVaMs are currently diagnosed most frequently by vascular ultrasound (vUS) comprising Color Coded Doppler sonography (CCDS) and Power Doppler (PD) but as well by magnetic resonance imaging tomography (MRI) or by computerized tomography (CT).

The emergence of contrast-enhanced ultrasound (CEUS) has exceptionally improved the ability to image the circulation in some disease circumstances (e.g. in confined liver lesions [6–12]) but without perfusion imaging quantification we are not able to interpret objectively the severity of the vascular impairment. Recently, we reported on the first CEUS analysis of 18 HHT cases concerning hepatic macro- and microcirculation, complementing qualitative results by quantitative perfusion time intensity curve (TIC) evaluation. Our findings showed significant distinctions in time to peak (TTP) and area under the curve (AUC) values in the four selected regions: hepatic artery, shunt region, portal vein and hepatic parenchyma [13].

To be able to sub-classify a high risk cohort of asymptomatic HHT patients with potential therapeutic indication, we improved the technical preconditions by implementation of motion adjustment in VueBox mode, and detection is fitted for every image to equalise for breathing artifacts. Moreover, we now examined a substantially bigger cohort of 34 patients (31 of them presenting HvAMs).

## Material and methods

### Patients

CEUS imaging information of 34 cases were retrospectively studied. Diagnostic analysis was made taking account of the Curaçao criteria. The database was browsed for every patient in the years 2015–2017 who has undergone CEUS screening after being diagnosed with HHT in the Ear-Nose-Throat (ENT) department of the University Hospital of Regensburg (UKR).

This investigation was approved specifically by the ethical committee of the UKR (approval number: 15-104-0233), all patients agreeing by written informed consent to injection of contrast agent for CEUS examination. The ethical committee waived the requirement for informed consent regarding the patient records used in this retrospective study.

### Imaging examinations

Each CEUS examination was performed with a high-end US scanner (LOGIQ E9, GE Healthcare, Milwaukee, USA). The frequency of the convex transducers straddled from 1.0 to 6.0 MHz, each being constructed for abdominal application. Contrast harmonic imaging (CHI) in the form of amplitude modulation (AM) or pulse inversion harmonic imaging (PIHI) was implemented in the US device. A sulfur hexafluoride-filled microbubble contrast agent (SonoVue, Bracco, Milan, Italy) was utilized in this study. A capacity of 1.0 to 2.4 ml of this agent was applied intravenously as a bolus in the antecubital vein, followed by application of 10 ml of 0.9% NaCl.

Each CEUS examination was conducted by one radiologist with more than five years of expert knowledge in CEUS and who evaluated more than 3000 US/year across more than 15 years. Homogenous imaging configurations were utilized and all the US examinations were conducted according to standard procedure. Complete screening of the liver was accomplished by B-Mode examination previous to CEUS for every patient. Then, using CCDS, flow parameters from the portal vein, the hepatic artery (center, right and left part of the liver) and the liver veins were documented. For CEUS a sweep technology was applied for assessment of contrast enhancement in the center and the peripheral parenchymal regions. The mechanical index (MI) was decreased below 0.2, which permits efficient tissue annulment to generate almost pure microbubble pics and impede their corrosion. CEUS operating mode and a chronograph were initiated simultaneously when contrast agent was administered. The CEUS clips until 120 s following application were recorded continously, neither in any change in the machine configurations nor movement of the tansducer. After 120 s the transducer was stired to examine the entire liver. For repeated evaluations Baseline US images and CEUS movie clips were stored digitally on the hard disks of the US device and transferred to an archiving software for evaluation.

### Image analysis

All US images and clips were analyzed retrospectively as stored DICOM by two impartial scientists who were not involved in the examination process and were uninformed about pertinent clinical, laboratorial, histopathological data and the results of other imaging techniques. Different opinions on the enhancement pattern and intensity were solved by consent. The results were examined concerning the proposed sonographic criteria by Caselitz [14], Buonamico [15] and Buscarini (EASL guidelines 2015) [16, 17] to prove the diagnosis of HVaMs in patients with HHT. Macro- and microshunts were documented if detected in the different liver segments.

The CEUS phase was subdivided into arterial phase (10–45 s from contrast agent administration), portal venous phase (45–120 s) and late phase (121–360 s) [7, 8, 10]. The intraductal enhancement extent was compared to the adjacent liver parenchyma and was classified into hyper-, iso-, hypo- and non-enhancement in accordance with the recently released guideline [6]. The enhancement structure was subclassified into homogeneous and heterogeneous. For repeated analysis, the FLASH dynamic evaluation of CEUS was used.

The digital stored DICOM cine loops (up to 1 min) were uploaded and opened for blinded and impartial evaluation by an external software in the VueBox (BRACCO, Italy) on a different computer. The development of VueBox diminishes current limitations and facilitates quantification in a standardized manner. VueBox is color-coded, off-line, general-purpose perfusion program for dynamic CEUS investigations that utilizes automatic in-plane movement adjustment [18]. Regions of interest were located by hand in the shunt region (ROI 2), hilar region (ROI 3) and hepatic parenchyma (ROI 4), while ROI 1 comprised the whole liver. The mean intensity within a ROI can be figured as a function of time, in the form of a TIC, which delineates the wash-in and wash-out of the contrast agent in the ROI. At any time during the capture, enhancement can be detected by calculating relative echo power values in ROIs that describes the lesion and reference tissue. It is thus feasible when using bolus injection to determine amplitude parameters, such as peak enhancement (PE) and wash-in area under the curve (WiAUC). In order to consider also time parameters, we evaluated also the wash-in perfusion index (WiPI) which is WiAUC/rise time (RT; time until peak enhancement of the contrast agent is reached). PE, WiAUC and WiPI were measured for up to 60 seconds. To better compare the parameter values, we calculated the percentage in relation to the shunt region (100%). Images were generated automatically and color-coded findings were saved as an Excel data sheet. VueBox reveals color-coded parametric maps of the chosen perfusion parameter, created by TIC measured for all pixels of the map. The chosen TIC parameter (e.g. PE) is transferred to an associated color value of a color bar covering the spectrum of values and displayed at the anatomical region of the corresponding image pixel.

**Fig 1:**
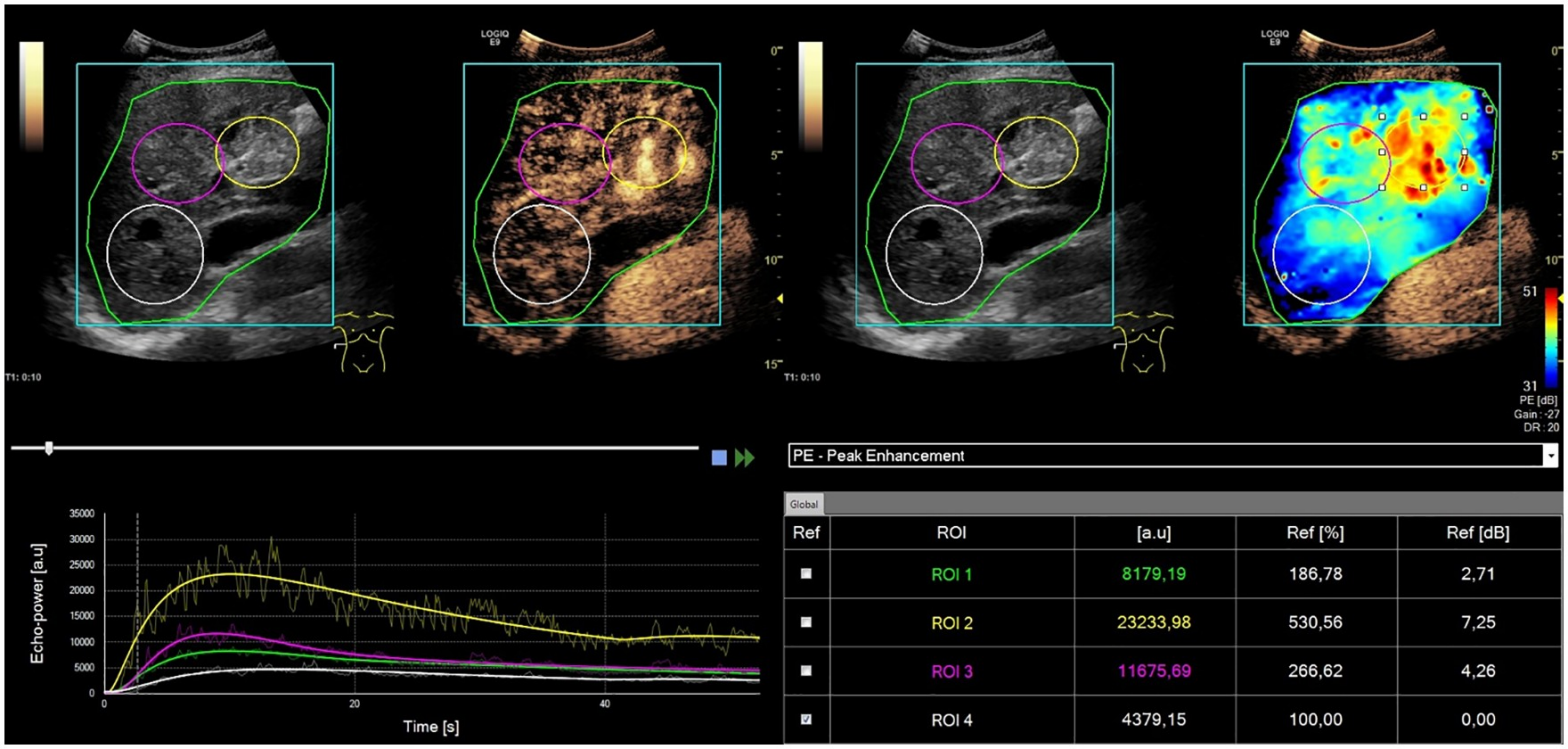
Arteriovenous hepatic malformations (shunts) in one exemplary case with HHT. **CEUS image and TIC-analysis illustrating peak enhancement**. Intrahepatic tortuos vascularization in segment IV and VIII of the liver being appreciable by early hyperenhancement in the course of the arterial phase following injection of 2.0 ml contrast agent. For TIC analysis three regions of interest (ROIs) were positioned in the shunt region (yellow), hilus region (purple) and hepatic parenchyma (white). TIC analysis demonstrated significant highest PE in the shunt region, next in the hilus and lowest in the hepatic parenchyma. The VueBox screen is segmented in four quadrants: the original image with the ROIs is showed in the upper left quadrant, the corresponding parametric image is depicted in the upper right quadrant, the corresponding TICs are displayed in the lower left quadrant in the color corresponding to the ROI in the image above and the numeric values of the chosen curve parameter is shown in the lower right quadrant.

The VueBox display is partitioned into four quadrants: the primordial image with the ROIs is posted in the upper left quadrant, the correlatively parametric image is shown in the upper right quadrant, the associated TICs [18] are displayed in the color analogous to the ROI in the image atop in the lower left quadrant and the associated quantitative values oft the chosen curve parameter are displayed in the lower right quadrant (Fig 1).

The VueBox can linearize and normalize intensity values acquired with various US devices, transducers and post-processing configurations, utilizing certain calibration files. Whereas the recorded cine clips were imported, the details oft the device and configuration that are utilized are inscribed from a preseted list of adjustments or read self-acting from the file header (ASR function disposable for certain devices). Calibration files for the US device and for the configuration that are utilized are supplied by Bracco [18, 19].

### Statistical analysis

The statistical analysis was performed applying Prism 6 software (Graphpad, La Jolla, CA, USA). The continuous values were indicated as mean ± standard error of the mean (SEM). The relation between numerical data was examined utilizing one- way ANOVA. P-values below 0,05 were considered statistically significant. Correlation between data was made by regression analysis. Regression formula and coefficient of determination (R^2^) is indicated for each diagram.

## Results

The patients were 11 men and 23 women, with a mean age of 59.3 years (range, 21–84 years) at the moment of HVaM diagnosis. HVaMs could be proved in 31 out of 34 patients. Malignant lesions were not detectable in any of the cases (Table 1).

**Table 1:** Basic characteristics of 34 patients with HHT.

case no.	gender (M/F)	age (years)	HVaMs (yes/no)	malignant lesions (yes/no)	group (1/2)	peak enhancement hilus/shunt (%)	portal vein diameter (cm)	portal vein peak velocity (cm/s)	Buscarini grading
**1**	**F**	**83**	**Yes**	**No**	**2**	**66.0**	**-**	**45**	**3**
**2**	**F**	**60**	**Yes**	**No**	**1**	**27.1**	**13.8**	**-**	**4**
**3**	**F**	**75**	**Yes**	**No**	**1**	**5.2**	**15.6**	**20**	**-**
**4**	**F**	**50**	**Yes**	**No**	**1**	**6.2**	**-**	**20**	**3**
**5**	**F**	**36**	**Yes**	**No**	**1**	**7.8**	**-**	**-**	**-**
**6**	**F**	**75**	**Yes**	**No**	**1**	**19.8**	**-**	**-**	**4**
**7**	**F**	**58**	**Yes**	**No**	**2**	**90.6**	**10.4**	**40**	**-**
**8**	**F**	**65**	**Yes**	**No**	**2**	**69.0**	**-**	**-**	**-**
**9**	**F**	**66**	**Yes**	**No**	**1**	**9.7**	**-**	**23**	**3**
**10**	**F**	**52**	**Yes**	**No**	**2**	**55.8**	**-**	**-**	**2**
**11**	**F**	**76**	**Yes**	**No**	**1**	**11.9**	**11.5**	**20**	**-**
**12**	**F**	**49**	**Yes**	**No**	**1**	**12.4**	**-**	**27**	**-**
**13**	**F**	**55**	**Yes**	**No**	**1**	**30.7**	**-**	**-**	**3**
**14**	**M**	**72**	**Yes**	**No**	**1**	**11.1**	**-**	**25**	**3**
**15**	**F**	**21**	**Yes**	**No**	**2**	**56.9**	**9.5**	**-**	**1**
**16**	**M**	**69**	**Yes**	**No**	**1**	**1.2**	**-**	**25**	**3**
**17**	**F**	**62**	**Yes**	**No**	**1**	**8.1**	**-**	**-**	**3**
**18**	**F**	**46**	**Yes**	**No**	**2**	**49.0**	**10.2**	**30**	**-**
**19**	**F**	**38**	**No**	**No**	**-**	**-**	**-**	**-**	**-**
**20**	**M**	**76**	**Yes**	**No**	**1**	**17.6**	**-**	**-**	**-**
**21**	**M**	**57**	**No**	**No**	**-**	**-**	**-**	**-**	**-**
**22**	**F**	**69**	**Yes**	**No**	**2**	**132.5**	**-**	**-**	**-**
**23**	**M**	**66**	**Yes**	**No**	**2**	**45.0**	**-**	**40**	**-**
**24**	**M**	**51**	**No**	**No**	**-**	**-**	**-**	**-**	**-**
**25**	**F**	**45**	**Yes**	**No**	**1**	**29.1**	**-**	**-**	**3**
**26**	**M**	**75**	**Yes**	**No**	**1**	**24.1**	**-**	**-**	**3**
**27**	**F**	**31**	**Yes**	**No**	**1**	**6.4**	**-**	**-**	**-**
**28**	**F**	**48**	**Yes**	**No**	**2**	**75.6**	**-**	**-**	**-**
**29**	**F**	**84**	**Yes**	**No**	**2**	**50.0**	**-**	**-**	**-**
**30**	**M**	**62**	**Yes**	**No**	**2**	**51.3**	**-**	**-**	**-**
**31**	**F**	**57**	**Yes**	**No**	**1**	**9.7**	**-**	**-**	**-**
**32**	**F**	**42**	**Yes**	**No**	**1**	**20.6**	**-**	**-**	**3**
**33**	**M**	**73**	**Yes**	**No**	**1**	**25.8**	**-**	**-**	**-**
**34**	**M**	**72**	**Yes**	**No**	**2**	**76.7**	**-**	**30**	**2**

**Fig 2:**
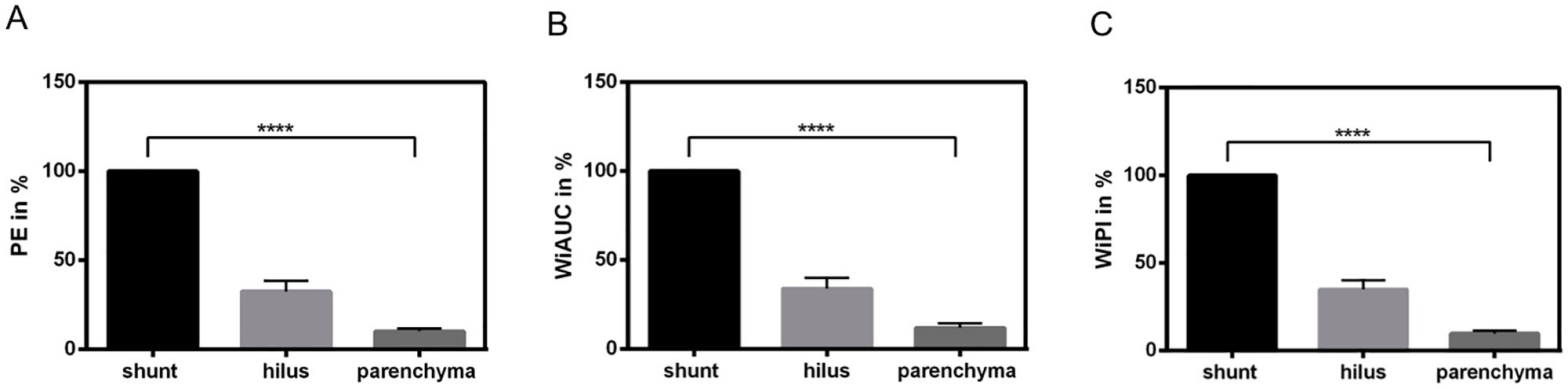
Quantitative perfusion data (TIC) analyzed by CEUS combinend with VueBox color-coded perfusion software within shunt region, hilus region and hepatic parenchyma in 31 patients with diagnosed HHT. Percentage in relation to PE (A), WiAUC (B) and WiPI (C) of the shunt region (100%). Marked significant distinctions (p<0.0001) in PE, WiAUC and WiPI values was identified between the three regions, to the effect that uppermost values were detected in the shunt region (100%), next up in the hilus (PE 32.6%; WiAUC 33.9%; WiPI 34.1%) and the lowest were demonstrated in the liver parenchyma (PE 10.2%; WiAUC 12.0%; WiPI 9.5%).

Marked significant differences (p < .0001) in all perfusion parameter values were identified between the three regions, to the effect that uppermost values were found in the shunt region (100%), next in the hilar region (PE 32.6%; WiAUC 33.9%; WiPI 34.1%), and the lowest in the hepatic parenchyma (PE 10.2%; WiAUC 12.0%; WiPI 9.5%). The relative differences between the regions were alike for PE, WIAUC and WiPI (Fig 2).

**Fig 3:**
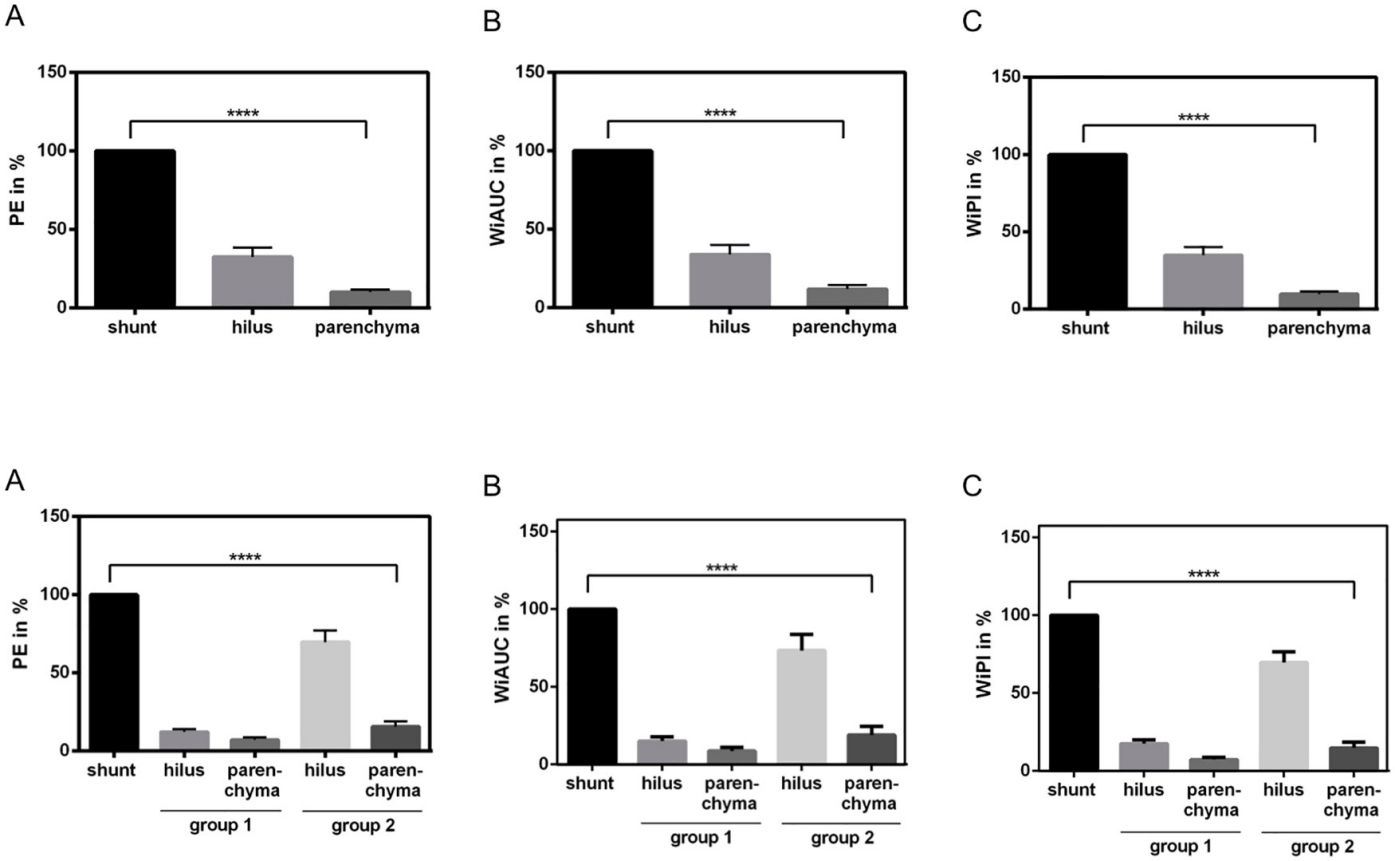
Subset analysis of quantitative perfusion data (TIC) evaluated by CEUS combinend with VueBox color-coded perfusion software within shunt region, hilus region and hepatic parenchyma in 31 patients with diagnosed HHT. Percentage in relation to PE (A), WiAUC (B) and WiPI (C) of the shunt region (100%). Marked significant distinctions (p<0.0001) in PE, WiAUC and WiPI values was identified between the two groups concerning the three selected regions, to the effect that in group 1 (hilus PE 12.1%; WiAUC 15.1%; WiPI 17.2%; liver parenchyma PE 7.2%; WiAUC 8.7%; WiPI 7.4%) obvious lower perfusion parameters could be demonstrated when compared to group 2 (hilus PE 69.8%; WiAUC 73.5%; WiPI 69.6%; liver parenchyma PE 15.5%; WiAUC 18.9%; WiPI 14.9%).

In our study, the perfusion parameter values varied among the patients but it was obviously that the relative difference between PE, WIAUC and WiPI values in the shunt region compared to hilus and hepatic parenchyma was dramatically and significantly increased in one group of patients (group 1: hilus PE 12.1%; WiAUC 15.1%; WiPI 17.2%; liver parenchyma PE 7.2%; WiAUC 8.7%; WiPI 7.4%) vs. the other (group 2: hilus PE 69.8%; WiAUC 73.5%; WiPI 69.6%; liver parenchyma PE 15.5%; WiAUC 18.9%; WiPI 14.9%). We choose 40% as cut-off value and the shunt region/hilus region ratio as decisive for distribution of patient data to group 1 respectively 2, this means that all patients with perfusion parameters lower than 40% in hilus region compared to shunt region were repartitioned to group 1, the other to group 2. Beyond the differences to the shunt region, in group 1 the hilus region and the hepatic parenchyma showed similar perfusion patterns, while in group 2 the hilar perfusion values were evidently higher compared to the liver parenchyma. Moreover, PE, WIAUC and WiPI in the hepatic parenchyma was decreased in group 1 compared to group 2 (Fig 3).

**Fig 4:**
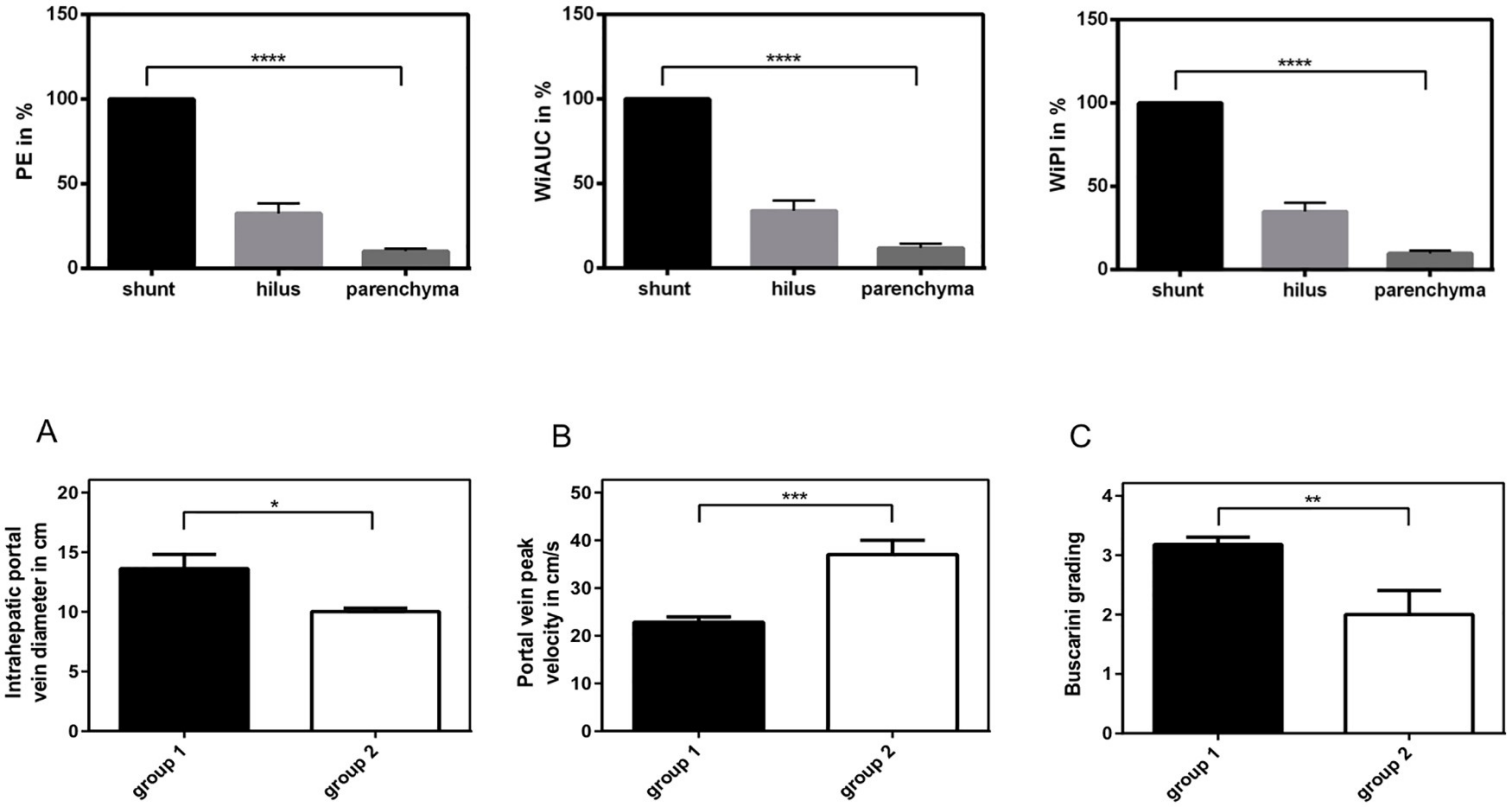
Clinical parameters in group 1 vs. group 2 of our patient cohort. Intrahepatic portal vein diameter in cm (A), portal vein peak velocity in cm/s (B) and Buscarini grading (C) in group 1 compared to group 2 of our patient cohort. Significant distinctions (A: p<0.05; B: p<0.001; C: p<0.01) was identified between the two groups, to the effect that in group 1 the intrahepatic portal vein diameter was significantly higher (13.6 cm vs. 10.0 cm; n = 3), while portal vein peak velocity was significantly lower (22.8 cm/s vs. 37.0 cm/s; n = 5–7) than in group 2. Moreover, Buscraini grading was significantly elevated in group 1 compared to group 2 (3.2 vs. 2.0; n = 4–11).

**Fig 5:**
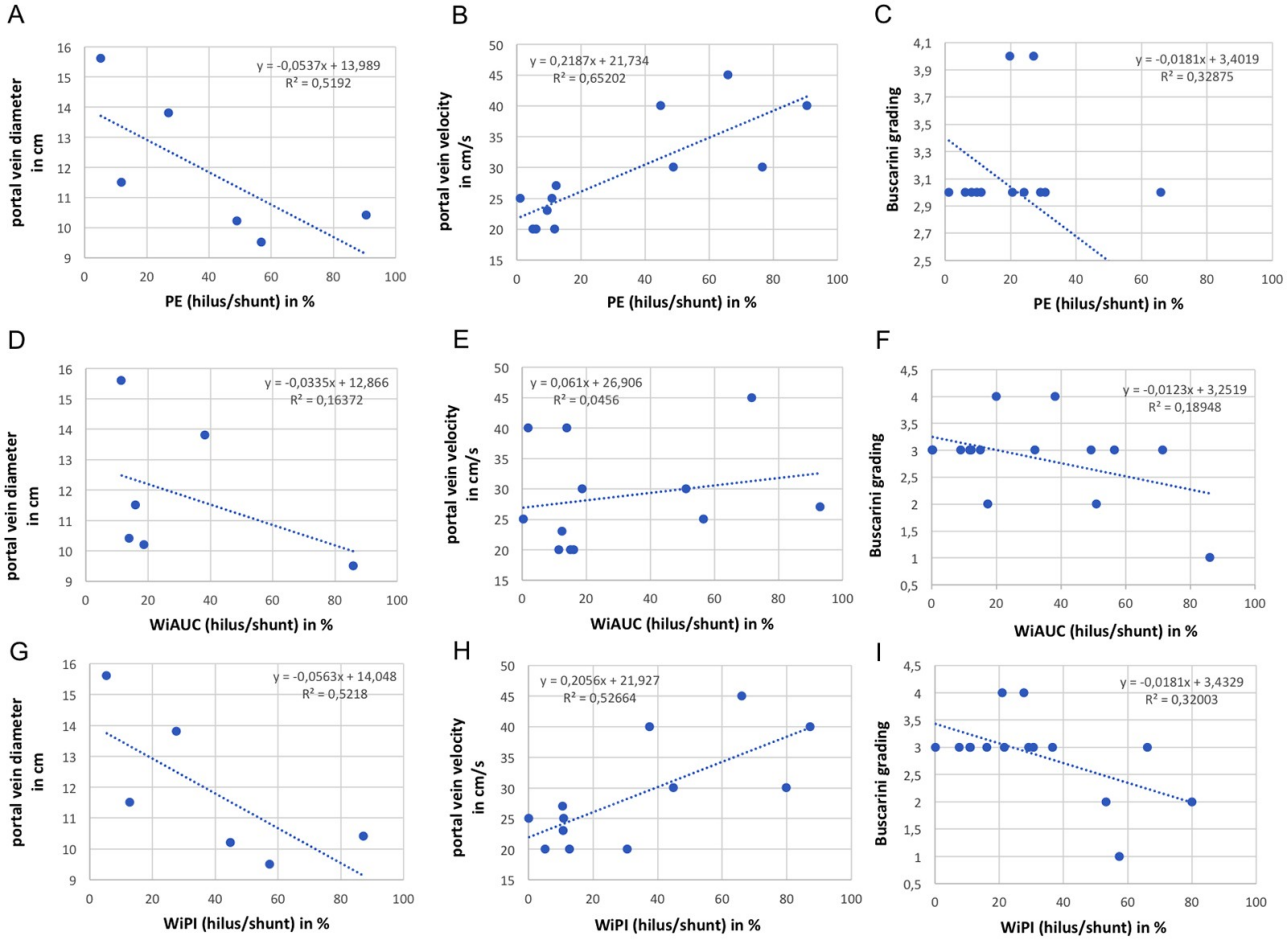
Correlation between clinical parameters and TIC parameters of our study cohort. Regression analysis showing the relationship between portal vein diameter (in cm), portal vein peak velocity (in cm/s), Buscarini grading and PE (A-C), WiAUC (D-F), WiPI(G-I) (each: hilus/shunt in %). Regression formula and coefficient of determination (R^2^) is indicated for each diagram.

Aditionally, we screened the patient files for clinical data to provide evidence for correlation to the parameter results. As shown in Fig 4, in group 1 the intrahepatic portal vein diameter was significantly higher (p<0.05), while portal vein peak velocity was significantly lower (p<0.001) than in group 2. Moreover, Buscraini grading was significantly elevated (p<0.01) in group 1 compared to group 2. By performing regression analysis (Fig 5), we could provide evidence for the association between these data and PE or WiPI, to the effect that the best correlation could be evidenced to portal vein peak velocity (PE: R^2^ = 0,65; WiPI: R^2^ = 0,53) and portal vein diameter (PE: R^2^ = 0,52; WiPI: R^2^ = 0,52) while the association to Buscarini grading was acceptable (PE: R^2^ = 0,33; WiPI: R^2^ = 0,32). Regarding WiAUC, regression analysis showed no correlation to portal vein diameter (R^2^ = 0,16), portal vein peak velocity (R^2^ = 0,05) or Buscarini grading (R^2^ = 0,19).

## Discussion

HHT is an uncommon autosomal dominantly transmitted disorder characterized by systemic vascular dilatation leading to arteriovenous fusion in the form of telangiectases or major vascular malformations, implying the chance of hemorrhagic incidents [20].

HVaMs can be diagnosed in the majority of HHT cases, however rarely (8%) being symptomatic [3, 21]. In symptomatic patients, identification of HVaMs is normally made by vUS comprising CCDS and PD but also likewise by MRI and CT. By latest attempts to discover particular risk factors for evolution to the symptomatic stadium and consequently to identify a high risk cohort of asymptomatic cases on the one hand and the confirmed usefullness of angiogenesis inhibitors like bevacizumab for therapy of HVaMs on the other hand, cost-effective examination of HHT patients for liver involvement get increasingly interesting [22].

In our latest study we evaluated for the first time HVaMs in a cohort of patients (n = 18) using CEUS and adding quantitative perfusion analysis. Our results revealed significant differences in TTP and AUC values in four selected regions: hepatic artery, shunt region, portal vein and hepatic parenchyma.

This time, we ameliorated the preconditions by implementation of the VueBox mode and by evaluation of a considerably bigger cohort of 34 patients. HVaMs could be ascertained in 91% of the patients, thus in higher percentage than in literature (78%) [3] and substantially increased compared to studies with only baseline US examinations (53%; [23]). In none of the cases malignant lesions developed. The shunts were located especially in segment 8 and the left hepatic lobe.

Performed by an expert, CEUS is a reasonable examination to investigate the dynamic microcirculation in liver disorders [19, 24]. For characterization of focal liver lesions, the digital resolution of CEUS is sometimes better than that of contrast-enhanced CT or MRI, where identification of early arterial phase enhancement is missed because of lower frame rates. Moreover, CEUS is a non-invasive, non-irradiating procedure with no cardio-, hepatic- or nephrotoxic effects [25]. Our study indicates that combination with VueBox color-coded perfusion software analysis could be applied for a critical evaluation of perfusion intensity in HHT patients with HVaMs. The software displays hyper-enhancement in yellow and red coloring, whereas devascularization showes up in blue. By applying ROIs in the shunt region, the hilar region and the liver parenchyma, the extent of perfusion intensity can be evaluated. It is widely accepted in current european clinical guidelines that clinical outcome correlates with the extent of perfusion dysregulation and that this permits a individual patient management and follow-up [17].

Our analyses revealed significant highest PE, WiAUC and WiPI in the shunt region, subsequently in the hilar region, and the lowest in the liver parenchyma. Interestingly, the relative differences between the regions were uniform for PE, WIAUC and WiPI, so that measuring of one parameter could be enough for diagnosis and follow-up of HHT.

One marker parameter could be PE which was implyed also by our group in the evaluation of successful treatment after percutaneous interventional procedures for liver tumors [26]. In our study, the PE parameters in the shunt region compared to hilus and hepatic parenchyma were significantly up to more than 10–fold elevated in one subset of patients compared to the other. Beyond, in subset 1, PE of the hilus region and the hepatic parenchyma were alike, whereas in group 2 the hilar PE values were distinctly elevated compared to the liver parenchyma. The PE values of the liver parenchyma are moreover decreased in subset 1 compared to 2. This could indicate, that in group 1 hepatic perfusion concentrates in the shunt region, neglecting the rest of liver, while subset 2 is not thus affected. The results for WiAUC and WiPI were similar to those for PE.

The group with markedly enhanced perfusion intensity could be the high risk cohort of HHT patients with the highest benefit regarding angiogenesis inhibitors like bevacizumab for HVaM treatment. To strengthen this presumption, we examined the available patient files for clinical information to provide evidence for linkage to the parameter findings. Portal hypertension is one of the most common complications of HHT. It is associated with higher portal vein diameter [27, 28] and lower portal vein velocity [29, 30] within the liver. As showed in Figs 1–3, in the estimated high-risk group 1 the intrahepatic portal vein diameter was significantly higher, while portal vein peak velocity was significantly lower than in group 2. Moreover, Buscraini grading was significantly elevated in group 1 compared to group 2, estimating worse outcome for this group of patients.

By regression analysis, we could provide evidence for the association between these data and PE or WiPI, while surprisingly WiAUC values could not be correlated to the above mentioned clinical parameters. Therefore, we conclude that not every TIC parameter which is significantly increased in the shunt region, compared to other regions of the liver, has obligatory clinical significance.

Of course, these presumptions have to be tested in prospective trials correlating CEUS findings with clinical parameters (liver parameters, high-output cardiac failure, portal hypertension, iron deficiency anemia). This will be also necessary for better selection of the perfusion parameter cut-off values when repartitioning them to the two groups. We arbitrary have chosen 40% hilus/shunt region as cut-off value in this study to show the existence of at least two distinct groups with different perfusion patterns.

Thus far, there are no contraindications for recurrent injections of SonoVue. Nevertheless, allergic responses towards this contrast agent can emerge [31]. The microbubbles rest stringent intravascular [32], therefore hepatic microcirculation can be examined in real-time with enhanced diagnostic precision [19, 33], and the most elevated site resolution of imaging techniques. Beyond, compared to other methods, CEUS is a cost-efficient technique [34].

A limitation is that VueBox is not incorporated in the US device. This sophisticated investigation needs both an exercised investigator and extra-equipage. The examination with VueBox is time consuming requiring nearly 30 minutes per examination.

A previous critical point, that a lot of ultrasound investigations cannot be evaluated because of movement and breathing artifacts, is eliminated by VueBox thanks to the incorporated motion compensation [35]. Using this software, quantification of CEUS images permits recognition of contrast enhancement over a broad range of concentrations, ameliorating the evaluation of minor perfused areas without oversaturation of the regions with normally perfusion pattern.

As another limitation of this study, information about clinical parameters was missing in many cases because the patients are mainly treated in hospitals far away from the University Hospital of Regensburg and are coming only for specialized evaluation in our ENT department and for CEUS examination in our US Center.

## Conclusions

The expertise about US properties of HVaMs augmented in the last years, particularly by applying CCDS- and PD-US but there is few knowledge about quantitative perfusion characteristics. Our study showed significant differences in PE, WiAUC and WiPI values in the three determined areas: shunt region, hilus and hepatic parenchyma. Besides, the relative perfusion parameter values in the shunt region compared to other areas were significantly elevated in one subset of patients. As to that, we could correlate PE and WiPI to clinical parameters (portal vein diameter, portal vein peak velocity, Buscarini grading). These novel results could be utilized to sub-classify a high risk cohort of asymptomatic patients with therapeutic indication. Moreover, with novel upcoming therapeutic modalities like angiogenesis inhibitor bevacizumab, treatment of HVaMs and their complications will get more appealing in future. Thus, CEUS investigation is able to fill the hole of necessary precise cost-effective screening methods in HHT patients with HVaMs.

## Supporting information

S1 Table The values (extracted from images) behind the means, standard deviations and other measures reported used to build graphs. (XLSX)
